# Genotyping-by-Sequencing SNP Identification for Crops without a Reference Genome: Using Transcriptome Based Mapping as an Alternative Strategy

**DOI:** 10.3389/fpls.2016.00777

**Published:** 2016-06-15

**Authors:** Cécile Berthouly-Salazar, Cédric Mariac, Marie Couderc, Juliette Pouzadoux, Jean-Baptiste Floc’h, Yves Vigouroux

**Affiliations:** UMR Diversité, Adaptation et Développement des Plantes, Institut de Recherche pour le DéveloppementMontpellier, France

**Keywords:** SNP, GBS, UNEAK, transcriptome, site frequency spectrum, pearl millet

## Abstract

Next-generation sequencing opens the way for genomic studies of diversity even for non-model crops and animals. Genome reduction techniques are becoming progressively more popular as they allow a fraction of the genome to be sequenced for multiple individuals and/or populations. These techniques are an efficient way to explore genome diversity in non-model crops and animals for which no reference genome is available. Genome reduction techniques emerged with the development of specific pipelines such as UNEAK (Universal Network Enabled Analysis Kit) and Stacks. However, even for non-model crops and animals, transcriptomes are easier to obtain, thereby making it possible to directly map reads. We investigate the direct use of transcriptome as an alternative strategy. Our specific objective was to compare SNPs obtained from the UNEAK pipeline as well as SNPs obtained by directly mapping genotyping-by-sequencing reads on a transcriptome. We assessed the feasibility of both SNP datasets, UNEAK and transcriptome mapping, to investigate the diversity of 91 samples of wild pearl millet sampled across its distribution area. Both approaches produced several tens of thousands of single nucleotide variants, but differed in the way the variants were identified, leading to differences in the frequency spectrum associated with marked differences in the assessment of diversity. Difference in the frequency spectrum significantly biased a large set of diversity analyses as well as detection of selection approaches. However, whatever the approach, we found very similar inference of genetic structure, with three major genetic groups from West, Central, and East Africa. For non-model crops, using transcriptome data as a reference is thus a particularly promising way to obtain a more thorough analysis of datasets generated using genome reduction techniques.

## Introduction

In the last two decades, next-generation sequencing (NGS) technologies (Mardis, 2008) have made the assembly of numerous new reference genomes possible (Ellegren, 2014). Yet, in the case of non-model organisms, accessing genome diversity remains a challenge. Sequencing only a fraction of a large genome has been proposed as a promising way of getting round this constraint (Narum et al., 2013). Reduced-representation library (RRL) sequencing approaches enable sequencing of a fraction of the genome as well as of homologous regions in a set of individuals. Among RRL techniques, two main approaches are widely used today: the RAD-seq approach (Baird et al., 2008; Davey et al., 2011) and the genotyping-by-sequencing (GBS) approach (Elshire et al., 2011) but several others are also available (e.g., PE-RAD, dd-RAD, 2b-RAD, ezRAD). GBS, like RAD-seq, reduces genome complexity through restriction digest, but offers a simplified and more cost-effective library preparation protocol (Elshire et al., 2011). These molecular techniques were developed at the same time as specific bioinformatics pipelines to handle the resulting NGS raw sequences. For instance, the Stacks pipeline was developed primarily for RAD-seq data (Catchen et al., 2011, 2013), while the TASSEL pipeline was developed for the GBS approach (Glaubitz et al., 2014).

Therefore, even though RAD-seq and GBS data can be analyzed using either pipeline, they are preferentially analyzed using their original corresponding pipeline. There is also a preference for each RRL approach that depends on the “scientific community” concerned. For instance, RAD-seq is widely used for evolutionary history and conservation studies on wild organisms (Hohenlohe et al., 2013; Pujolar et al., 2014; Combosch and Vollmer, 2015), whereas GBS is used by researchers working on crops and domesticated animals. The TASSEL pipeline was thus primarily developed to handle low coverage sequencing for homozygote samples (Glaubitz et al., 2014) and to be used in genome wide association studies (Moumouni et al., 2015; Sonah et al., 2015; Upadhyaya et al., 2015). Even among crops, not all species are model organisms with a reference genome. When no reference is available, somewhat similar strategies are implemented in Stacks and TASSEL to identify SNPs. First, similar reads are identified and grouped together to create TAGs. Second, networks of TAGs are built to identify which TAGs could be considered as alternative copies of the same genomic loci. These steps depend on several parameters, such as minimum coverage, for a read to be considered as a TAG, or the number of mismatches between two TAGs to be considered as alternative copies of one locus or different loci. The TASSEL “no reference genome” pipeline is implemented in the UNEAK (Universal Network Enabled Analysis Kit) module (Lu et al., 2013). SNPs are identified by drawing simple networks of reciprocal TAGs that only differ by 1 bp mismatch. Significant effects of pipeline parameters on SNPs identified and population genetics inferences have been highlighted for Stacks (Catchen et al., 2013; Mastretta-Yanes et al., 2014; Rodríguez-Ezpeleta et al., 2016). To our knowledge, the effects of the UNEAK calling approach on population genetics have not yet been investigated.

An alternative strategy would be to map genomic reads from RRL approaches directly on a transcriptome. Most non-model crops possess a transcriptome reference that was primarily built for transcriptome studies. While building a transcriptome was formerly challenging (Martin and Wang, 2011; Góngora-Castillo and Buell, 2013), new tools are available today that make it possible to rapidly and efficiently obtain a new assembly (Grabherr et al., 2011). Transcriptomes enable access to longer sequences around SNPs, a very interesting feature for further SNP validation and access to an annotation of the genomic region. Thus, using a transcriptome reference to map reads from RRL approaches (Russell et al., 2013; Combosch and Vollmer, 2015) could be an interesting alternative for SNP discovery.

However, it is not easy to assess the bias arising from using the SNP calling pipeline, especially for population genetic studies (Hohenlohe et al., 2010; Nielsen et al., 2012; Arnold et al., 2013; Davey et al., 2013; Gautier et al., 2013; Han et al., 2014; Ilut et al., 2014; Harvey et al., 2015; Rodríguez-Ezpeleta et al., 2016). Therefore, in the following, we compare two sets of SNPs obtained from wild pearl millet populations using GBS sequencing. The first set of SNPs was obtained through the UNEAK pipeline without a reference genome and the second set was obtained through a mapping pipeline to the pearl millet transcriptome. We therefore investigated the differences and congruence in SNPs called for the assessment of population structure and analysis of genetic diversity.

## Materials and Methods

### Plant Material

**FIGURE 1:**
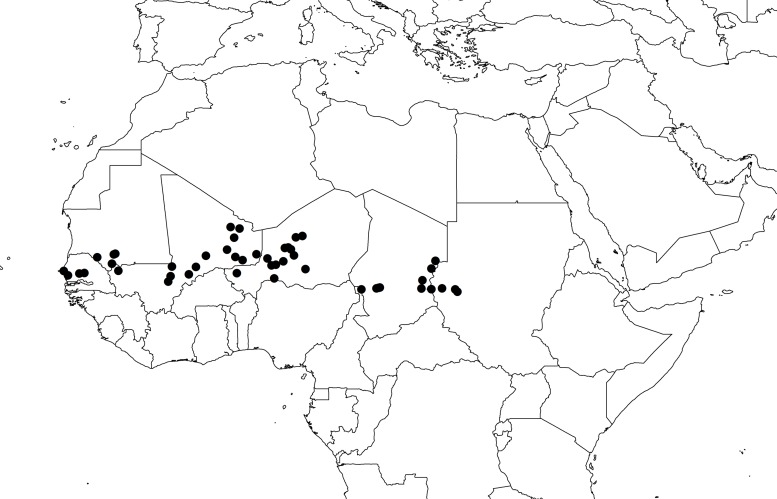
**Geographical distribution of the 48 populations of wild pearl millet**.

We selected 48 wild pearl millet populations [*Pennisetum glaucum* (L.) R. Br. ssp. *monodii*] from a collection held at IRD (Institut de Recherche pour le Développement, Montpellier, France). The 48 populations were chosen to cover the known distribution of wild pearl millet (**Figure 1**). Seeds were grown in the greenhouse until flowering, and inflorescences from 10 plants per population were collected for DNA extraction. DNA was extracted using the MATAB protocol (a modified CTAB/β-mercaptoethanol method; Mariac et al., 2006). A set of 95 DNA normalized to 100 ng/μl (sample size per population ≤ 2) was sent to the Institute for Genomic Diversity at Cornell University^1^ for GBS genotyping. Details on GBS protocol details can be found elsewhere (Elshire et al., 2011; Cronn et al., 2012). Genomic libraries were constructed using *ApeKI* restriction enzyme. The resulting 95-plex library was sequenced with an Illumina HiSeq2000. Four samples were not used for subsequent analyses due to the high rate of missing genotypes (>70%).

### SNP Discovery and Genotype Calling

#### UNEAK Pipeline

Raw sequences were processed with a modification of the TASSEL-GBS pipeline (Glaubitz et al., 2014): the UNEAK pipeline (Lu et al., 2013). With the UNEAK pipeline, the alignment of TAGs to a reference genome is replaced by the creation of a pair of TAGs and network filtering to enable SNP discovery (Lu et al., 2013). Briefly, good reads were defined as reads carrying a perfect barcode match with no Ns in the 64 bp following the barcode. Reads were subsequently trimmed to 64 bp (excluding barcodes). Unique 64-bp sequence TAGs that were present five or more times across all samples were retained and used to identify “TAG pairs,” with a default error tolerance rate (ETR) of 0.03, as described in Lu et al. (2013). Reciprocal “TAG pairs” with only 1 bp mismatch were considered as putative SNPs. Likelihood scores for each possible genotype were calculated according to formula 3.8 of Etter et al. (2011) and the most likely genotype was assigned. SNPs with a minor allele frequency (MAF) below 0.05 were excluded. Analyses were conducted with TASSEL version 3.0.157. The final set of SNPs (262,928) was then filtered for depth of coverage (DP) and for the percentage of missing data per SNP (<10%). We use the median value of coverage across all the SNP as threshold for the DP filter.

#### Transcriptome Based Mapping (TM) Pipeline

The wild pearl millet transcriptome contains 50,313 contigs for a total of 36.5 MB. This transcriptome was built from RNA from early inflorescences when differential expression was not too pronounced. The average contig length is 725 bp ± 732 bp (the transcriptome assembly^2^).

Raw sequences were first trimmed for low quality ends (<20) and reads of less 35 bp were removed using Cutadapt 1.2.1 (Martin, 2011). Secondly, a filter on read mean quality was applied at a threshold of 30. Reads were mapped to the assembly with BWA version 0.7.5 (Li and Durbin, 2009) with –n 3, allowing for a maximum number of three mismatches. Unmapped reads were removed using SAMtools version 0.1.17 (Li et al., 2009). We used RealignerTargetCreator and IndelRealigner from GATK version 2.4.7 (DePristo et al., 2011) to handle indels. SNPs and genotypes were called using UnifiedGenotyper. A total of 236,897 SNPs were then filtered for no more than three mismatches per 10 bp window, a HARD_TO_VALIDATE mapping quality (MQ) filter was applied [MQ0 ≥ 4 && ((MQ0/(1.0 ^∗^ DP)) > 0.1], and filtering was performed for QUAL (Quality) and QD (Quality by Depth) parameters which derived from Illumina quality scores (QUAL ≤ 60; QD ≤ 6.87 quantile 5%). The 121,279 remaining SNPs were then filtered for DP using the median value, and the percentage of missing data per SNP (≤10%). It is important to note that the additional quality filters cannot be applied in the UNEAK pipeline since Illumina quality scores are not used and not kept through the pipeline. All command lines are available in Supplementary Data File S1, and datsetes are availbale at https://sites.google.com/site/africropproject/data.

#### Overlap between the Two SNP Datasets

We aligned the Hapmap file of TAG sequences on the transcriptome using BWA version 0.7.5 (Li and Durbin, 2009) with –n 3, allowing for a maximum number of three alignments to output. We only report TAGs that had a unique hit.

In order to identify SNPs shared by the two datasets, we identified TAGs among the 21,913 final UNEAK SNPs that aligned to the transcriptome and extract the SNP position. We then compared the position and the alleles to identify homolog SNPs in the TM dataset.

### Diversity Statistics and Population Genetics Structure

We performed most analyses in the R environment (R Core Team, 2015^3^). We performed a principal component analysis (PCA) using SMARTPCA (Patterson et al., 2006; Price et al., 2006) as implemented in the R package SNPRelate (Zheng et al., 2012). We used the R package Adegenet (Jombart and Ahmed, 2011) to estimate heterozygosity values, and the R package Pegas (Paradis, 2010) for F-statistics. We used the sNMF software to identify population structure (Frichot et al., 2014). This software gives similar results to those obtained with STRUCTURE (Pritchard et al., 2000) but it is much faster and can handle a very large number of SNPs. Finally, the folded site frequency spectrum (SFS) was calculated and used to estimate Θ_w_, Θ_π_ and Tajima’s *D* (Tajima, 1989). In addition, we estimated the SFS expected for a population at equilibrium in each dataset (Fu, 1995).

## Results

### Mapping and SNP Discovery

**FIGURE 2:**
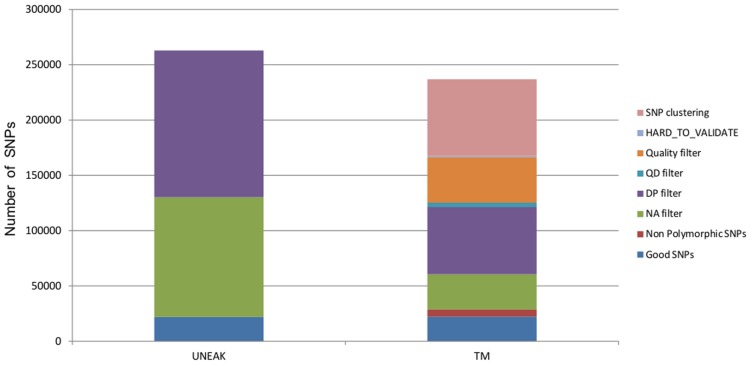
**Proportions of SNPs removed for each filter applied for both datasets UNEAK and TM**.

Both pipelines produced a similarly high number of SNPs. With the UNEAK pipeline, we were able to identify 262,928 biallelic SNPs. After filtering for depth (DP ≤ 51, 50.5% filtered) and missing data (NA ≥ 0.1, 41.2% filtered), we obtained 21,913 good quality SNPs. With the TM approach, a total of 16,399,078 cleaned reads with a mean size of 92 bp mapped on 36 918 contigs. The mean coverage was 41.33 ± 44.2 and the mean MQ was 24.5. We identified 238,897 biallelic SNPs with a median depth of 90×, after filtering we obtained a total of 22,262 good quality SNPs. Specific filters (SNP clustering, mapping and quality filters) from TM pipeline removed nearly 50% of SNPs, while subsequent filters for depth (DP ≤ 90) SNPs and missing data (NA ≥ 0.1) removed 25 and 13.5%, respectively (**Figure 2**).

The final sets of SNPs revealed that the quality of the two approaches was equivalent. The UNEAK final set of 21,913 SNPs had a mean DP per site and per sample of 7.24 ± 3.63 sd and an average missing rate per sample of 0.04 ± 0.04 sd. The TM final set of 22,262 SNPs had a mean DP per site and per sample of 8.68 ± 12 sd and an average missing rate per sample of 0.03 ± 0.03 sd. Within the TM final set, 56% of SNPs were found within a distance of 64 bp. The missing rates per sample between the UNEAK and TM dataset were highly correlated (*r* = 0.95). However, we had an average of 70% inflate number of missing data with UNEAK, since the average missing rates UNEAK:TM ratio was 1.7 ± 1.8 sd.

In addition, we tested direct mapping of the 262,928 UNEAK TAG 64 bp on the transcriptome. A total of 21,410 TAG loci (8%) mapped on 13,177 transcriptome contigs (26%). The mapping was relatively good since 94% of the mapped TAGs had a unique hit, among which 96% had a perfect 64 bp match. The mean MQ of these unique hits was 34 ± 9 sd. Among the 21,943 good quality TAGs, we found 3,146 TAGS (14%) that had good alignment on 2,382 (5%) contigs. Among those, we retrieved 822 SNPs common to the two datasets. Nearly all UNEAK SNPs had a MAF > 0.05 (**Supplementary Figure S1** and **Table S1**). The correlation coefficient between allele frequencies estimated by both pipelines for shared SNPs was very strong (*r* = 0.98).

### Genetic Structure and Genetic Diversity

**FIGURE 3:**
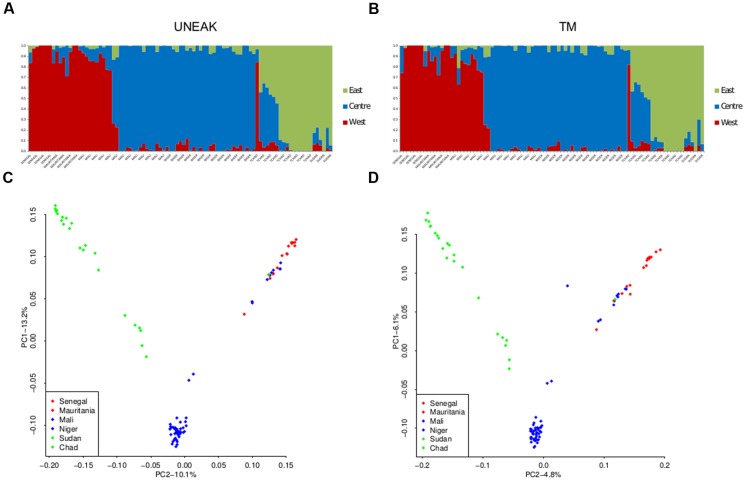
**Population structure inferences for both SNPs datasets UNEAK (left) and TM (right) using sNMF software (A,B) and PCA (C,D).** Western cluster is in red, Central cluster is in blue, and Eastern cluster in green.

The two datasets showed very similar inference of genetic structure. We identified *K* = 3 grouping populations geographically in a Western, Center, and Eastern clusters with both datasets (**Figure 3**). Correlations between admixture values from both approaches within each cluster were high with *r* > 0.99. The results of a PCA were similar (**Figure 3**). Both datasets showed the same three geographic clusters and the correlation between PCA coordinates was very high (*r* > 0.99). Comparing UNEAK and TM PCA, only one sample (sample 5726B1) was in a different position in the two plots. This individual had 17 times more missing data with the UNEAK dataset than with the TM dataset despite missing rates <0.05%. This very high ratio of missing data between datasets might explain its outlier status. More generally, the regression of PCA coordinates between the two pipelines showed that most individuals qualified as slight outliers had three times more missing data in the UNEAK pipeline than in the TM pipeline. However, overall, we observed very good individual quality and a very strong congruent inference of population structure irrespective which pipeline was used.

**FIGURE 4:**
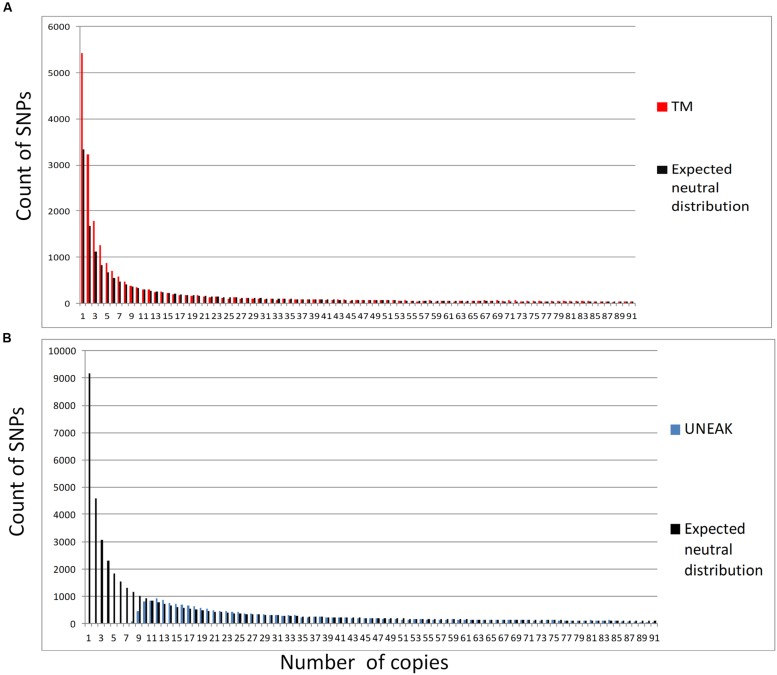
**Folded sites frequency spectrum for both SNP datasets: (A) TM (in red) and its expected neutral SFS in black; (B) UNEAK (in blue) and its expected neutral SFS in black**.

In contrast, genetic diversity assessment was affected differently depending on the pipeline. Heterozygosity values were almost two times higher with the UNEAK dataset than with the TM dataset (**Table 1**). For F-statistics, *F*_IS_ was slightly but significantly higher with the TM dataset and *F*_ST_ was significantly (two times) lower. When we compared observed SFS and expected SFS for a population at equilibrium, the UNEAK dataset clearly did not retrieve the expected amount of low frequency SNPs (**Figure 4**). On the other hand, TM SFS appeared to overestimate their number. As a result, Θ_π_ was 2.2 times higher with the UNEAK dataset and Tajima’s *D*-values consequently differed considerably with a positive Tajima’s *D*-value of 2.74 for UNEAK and negative value of –0.65 for TM dataset.

**Table 1:** Summary of diversity statistics for the two SNPs datasets.

	UNEAK			TM			*P*-value
	Mean	Median	Standard deviation	Mean	Median	Standard deviation	
H_Exp_	0.28	0.25	0.13	0.12	0.04	0.15	<0.0001
H_Obs_	0.16	0.13	0.13	0.09	0.03	0.14	<0.0001
*F*_ST_	0.22	0.2	0.18	0.1	0.04	0.18	<0.0001
*F*_IS_	0.34	0.37	0.28	0.39	0.44	0.4	<0.0001

## Discussion

In this study, we compared two bioinformatics pipelines and their impact on population genetics statistics. Investigating genomic diversity is still challenging for non-model organisms with large genomes. RRL sequencing approaches, such as RNA-seq and GBS approaches, have been proposed to reduce genome complexity. NGS data obtained can be handled by different pipelines including Stacks and TASSEL. Here, we preferentially used the TASSEL pipeline because is the most commonly used pipeline for crops studies. We therefore first used the UNEAK approach implemented in TASSEL and proposed and tested an alternative strategy in which NGS genomic reads were directly mapped on the pearl millet transcriptome. This strategy was guided by the observation that species transcriptomes are becoming progressively more accessible thanks to transcriptional studies and that it would be advantageous to use it (Russell et al., 2013; Combosch and Vollmer, 2015). It makes it possible to avoid using the *de novo* DNA assembly and has the advantage of using a reference genome, for example to access a longer sequence around SNPs sites, and has a greater probability of finding selection targets (Hancock et al., 2011).

### GBS Reads Biased Toward Coding Regions

The quality of our two final datasets is as good as the datasets used in other population genetic studies with final coverage ranging from 5 to 10 and missing values rates below 0.3. Many RRL datasets may have low coverage in studies whose design aims for more individuals or loci to increase the accuracy of population genetic parameters (Alex Buerkle and Gompert, 2013).

Surprisingly, we found that non-negligible numbers of UNEAK TAGs mapped to the transcriptome. Combosch and Vollmer (2015) found that about 15% of RAD loci mapped to 10% of transcriptome contigs. Our results are similar with 8% of TAG loci that mapped to 26% of transcriptome contigs. We originally expected a very low mapping rate since we were only mapping to the expressed genome. One possible explanation is the choice of the restriction enzyme used. Our study, and many others, used *ApeKI* enzyme with the GBS approach (Elshire et al., 2011; Lu et al., 2013). Its methylation sensitivity made it possible to eliminate repetitive methylated genomics regions from the experiment (Sonah et al., 2013). In eukaryotes, non-methylated sites are preferentially found in coding regions (Phillips, 2008). In *Populus* populations, 27% of restriction sites from the whole genome were recovered using *ApeKI* for GBS and of which 70% fell into annotated genes (Schilling et al., 2014). In sweet cherry (*Prunus avium* L.) 66% of SNPs were found in genic regions (Guajardo et al., 2015). In the present study, based on a pearl millet genome estimated at 1.8 G (Xin Liu, BGI, personal communication) and the size of the reference transcriptome at 36.5 MB, we interrogated only 2% of the genome. We found that 6–7% of reads per sample mapped to the transcriptome reference and 8% of UNEAK TAG loci were also aligned, which is three to four times more than the expected 2%. These results are in line with reports of an *ApeKI* enzyme bias toward coding regions in previous studies (Schilling et al., 2014; Guajardo et al., 2015).

### Effect of Pipelines on SNPs Identified

Pipeline specifics influence the number of SNPs discovered and their distribution properties. There are major differences in how SNPs are called between pipelines, because pipelines deal somewhat differently with sequencing errors, base quality values, SNP calling and genotype calling methods and in our case, TAG catalog construction vs. transcriptome mapping. We now review some of the differences between the two approaches and how such differences could impact our results.

Among all the parameters that can affect SNPs discovery, coverage is one of the most important. For instance, error rates are expected to increase with low coverage (<20×; Andrews and Luikart, 2014). To limit the impact of coverage in both our pipelines, we filtered SNPs with a depth above the median value for each dataset (51× for UNEAK, 90×for TM). Both final datasets had similar coverage and similar missing rates. Thus, it that sense, it would have little effect on number of SNPs discovered and population genetics estimates between datasets.

Another possible bias comes from repetitive regions in the genome, such as paralogs, and is not always easy to identify with NGS data. Different filters can be used to reduce the effect of unidentified paralogs. Paralogous regions are expected to align to multiple locations in the genome (Hohenlohe et al., 2012) and SNPs within paralogs genes are expected to show more than two alleles (Freedman et al., 2014). We only considered biallelic loci in the two datasets, since in RRL approaches, the problem of paralogs can be effectively addressed by ploidy-based filtering (Ilut et al., 2014). With the TM approach, we were able to apply an additional filter on MQ to reduce paralogous regions. However when mapping UNEAK TAGs to the transcriptome, we found that 94% of the TAGs that had a hit, mapped to a unique position. This suggests that even if no mapping filter can be applied, the probability of calling paralogs with the UNEAK pipeline is relatively low and the ploidy-based filtering thus appears to be sufficient to avoid paralog bias.

Statistical treatment of NGS sequences for a given genotype is based on assumed independent drawn of non-redundant read at a single gene. Several artifacts could bias the genotype likelihood because reads do not behave like the underlying statistical hypothesis: one read could be a duplicate (non-independent), an alternative allele could be missing (non-random draw) or mapping from two different but similar genes (not a single gene) on a single reference. Neither pipeline deals very easily with the occurrence of statistical non-independence of reads. Pipelines developed for RRL approaches were not able to handle allelic dropouts and mistake heterozygous presence/absence for homozygous presence/absence (Davey et al., 2013). A very recent pipeline for handling dominant and codominant markers has been developed (Fu et al., 2013). Yet with both of our approaches, a dominant marker (i.e., a mutation at the restriction site leading to allelic dropout) would have led to a homozygote call. Duplicate reads occur when, during DNA bank preparation, two reads derive from a single DNA by PCR duplication. PCR duplicates are by definition reads starting at the exact same mapping position. The effects of PCR duplicates on the estimation of population genetics have already been discussed (Arnold et al., 2013; Davey et al., 2013; Gautier et al., 2013). By construction, in RRL based on restriction enzymes, reads will start at the same mapping position which is the RE site, therefore applying PCR duplicates filter will not be possible unless a paired-end sequencing approach and random sheering is used (Davey et al., 2013) but recently a new protocol has been proposed by introducing “adaptor tags” allowing PCR duplicate discrimination (Tin et al., 2015). In conclusion, for both approaches we used, filtering for PCR duplicates was not possible and we therefore expected both UNEAK and TM datasets to underestimate heterozygosities. This is congruent with the strong correlation observed between estimated frequencies by both approaches for the shared SNPs.

The amount of SNPs allowed within a genomic windows is important since regions with too many SNPs are not reliable and may (i) contain many sequencing errors, (ii) be associated with paralogs. Within the TM pipeline, we applied a SNP clustering filter with no more than three SNPs per 10 bp. Nevertheless, it allowed quite a number of SNPs in a 100 bp read. For instance in the TM datasets, 56% of SNPs were less than 64 pb away. Since the UNEAK approach only allows 1 SNP per 64 pb, more than 50% of TM SNPs would be automatically discarded by the UNEAK pipeline.

However, the two pipelines differ strongly in their rare variant calling rates. Even if base quality is higher than 30 with the ILLUMINA sequencing platform, i.e., one error every 103 bases, with the amount of data that was generated, it ended up creating numerous errors. Calling rare variants (or not) will depend on the SNP and genotype calling algorithm implemented in the software and on how error sequencing rates are considered (Han et al., 2014). With some pipelines, the error rate estimate is considered to be constant across the genome, while other pipelines estimate an error rate for each base (Hohenlohe et al., 2011). Error rate estimates can also account for dependency between sequencing errors, (or not; Han et al., 2014). In GATK software, it is assumed that sequencing errors are independent and it takes coverage and base quality into consideration. Thus, unless coverage is about 10× per site per sample, GATK with UnifiedGenotyper can underestimate rare variants (Han et al., 2014), whereas UNEAK handles sequencing errors differently. To deal with this issue, the UNEAK pipeline uses a minimum ETR of 3% to call variants. This ETR has a direct impact on true low frequency variants: with erroneous SNP, true SNP are discarded. This way of handling the error sequencing rate might be the main reason why UNEAK SFS underestimates low frequency SNPs compared to the expected distribution with a population at equilibrium. It would also explain why so few SNPs are shared, since only frequent SNPs can be found by both datasets, which was confirmed by the distribution of MAF observed for shared SNPs.

In summary, we identified two main reasons for the low number of shared SNPs: (i) the constraint of no more than one SNP within 64 bp, and (ii) the uncovering of rare variants by UNEAK, which represent the majority of the polymorphism expected for a population at equilibrium. We ended up with relatively few shared SNPs. However, the allele frequency correlation between these SNP was very high.

### Effect of Pipelines on Diversity Estimates

How the specific characteristics of the SNPs we identified will affect population genetics estimates is another important question. There is an increasing literature on how parameters such as the number of mismatches allowed to assemble reads in orthologous loci with RRL approaches will influence the number of SNPs identified and population results. Most available studies focus on the effect of Stacks pipeline parameters (Catchen et al., 2013; Mastretta-Yanes et al., 2014). For instance, allowing a small number of mismatches would lead to the creation of more loci than in real life, and conversely, allowing too many mismatches would lead to merging paralogs. Being too stringent can increase genotyping error rates (Mastretta-Yanes et al., 2014) and overestimate homozygosity (Ilut et al., 2014). This could also have an effect on the identification of population structure (Harvey et al., 2015; Rodríguez-Ezpeleta et al., 2016). With the UNEAK pipeline only allowing 1 bp mismatch, it is the maximum stringency level for an RRL pipeline. Yet, we saw no effect on population structure and we observed very high congruence in the population structure in the two datasets and with two different methods: a Bayesian method and a PCA. These results are similar to those obtained by Rodríguez-Ezpeleta et al. (2016).

The main difference we observed between pipelines concerned the identification of low frequency variants. We found that the UNEAK pipeline was not able to recover rare variants while the SFS pattern for frequent variants was similar between pipelines. Thus methods based on “more frequent alleles” such as population structure approaches led to similar results. On the other hand, several statistics using low frequency variants differed considerably depending on the dataset used. Tajima’s *D* test (Tajima, 1989) is based on the SFS pattern, where an excess of rare variants is the sign of a population expansion or positive selection and inversely, a reduction in rare variants is the sign of a population contraction or balancing selection. Both pipelines gave highly contrasted results ranging from an overall negative value signature of –0.65 to a positive value signature of 2.74. Unbiased SFS is crucial for population genetics. Methods used to investigate population history including bottlenecks or expansion events are based on the difference between allelic diversity and heterozygosity and therefore depend on the identification of rare variants. Moreover, SFSs are widely used to test signatures of selection using Tajima’s *D* but so are other tests such as the CLR test (Nielsen et al., 2009). With such tests based on SFS, calling pipelines might significantly affect genomic regions found to be under selection.

Differences in the number of rare variants detected will also influence F-statistics in addition to heterozygosities. *F*_ST_ is dependent on allele frequency, low *F*_ST_ is expected for low frequency variants. Consequently, integrating more rare variants ends up adding low *F*_ST_ value, and thus lowering the mean *F*_ST_ value. The first and most simple consequence will be to make it difficult to compare diversity estimates obtained with different pipelines, an important issue in comparative studies (Ilut et al., 2014; Harvey et al., 2015). Another very “in vogue” approach since the NGS area, is the *F*_ST_ outlier detection approach for discovery of genes under selection. A number of *F*_ST_ outlier tests have been developed and extensively used for the discovery of candidate genes (Beaumont and Nichols, 1996; Vitalis et al., 2001; Beaumont and Balding, 2004; Foll and Gaggiotti, 2008; Bonhomme et al., 2010; Günther and Coop, 2013; Duforet-Frebourg et al., 2014) and are based on the expected distribution of *F*_ST_. Underestimating rare variants will affect the overall distribution of *F*_ST_ and might therefore have an impact on these selection tests.

It is certain that some of the differences in the results of diversity estimates observed between datasets are due to the fact that the UNEAK pipeline interrogates coding and non-coding regions while the TM pipeline only interrogates coding regions. However, like other authors, we previously observed that using *APEKI* biased SNP discovery toward coding regions (Combosch and Vollmer, 2015; Guajardo et al., 2015). All in all, we believe that the bias in the diversity estimates is mainly the result of the properties of the pipelines. Biased SFS are the result of different parameters including error rate estimation formula and the stringency allowed for TAG merging. An increasing number of studies suggest that genotype calling might no longer be needed for NGS data (Nielsen et al., 2011; Fumagalli et al., 2013; Han et al., 2014). Nielsen et al. (2011) pointed out that, until now, no satisfactory genotype calling algorithm is available that would lead to an unbiased SFS. These authors proposed a direct approach implemented in ANGSD software (Korneliussen et al., 2014) that does not intend to call genotypes and this approach has been extended by a modified PCA (Fumagalli et al., 2013) and admixture estimate approach based on genotype likelihoods (Skotte et al., 2013). However, this software works only with BAM files as input and a reference. Given this limitation, SNPs obtained from the UNEAK pipeline could not be used, whereas our TM pipeline could integrate such analysis.

## Conclusion

We have demonstrated the possibilities and discussed the advantages and disadvantages of two pipelines used for SNP discovery when no genome reference is available. We found that the UNEAK pipeline, with little and simple bioinformatics work, can efficiently identify a large number of SNPs as well as highlight genetic clustering. However, we observed notable underestimation of rare variants that could impact the estimation of population genetics and the detection of selection. Therefore, we encourage researchers to pay more attention to SFS. The transcriptome mapping reference was less biased in that sense and, more importantly, such a strategy could be used in combination with ongoing approaches without genotype calling to further reduce bias on SFS. The alternative strategy has the further advantage of enabling access to sequences surrounding SNPs for further genomic exploration. Moreover, since few SNPs are shared, both datasets could be combined, thereby significantly increasing the SNPs used.

## Author Contributions

CB-S, CM, and YV designed the project. CM, MC, and JP carried out the molecular laboratory work. CB-S and J-BF analyzed the data. CB-S and YV wrote the manuscript. All the authors discussed the results and commented on the manuscript.

## Conflict of Interest Statement

The authors declare that the research was conducted in the absence of any commercial or financial relationships that could be construed as a potential conflict of interest.

## Footnotes

1 http://www.biotech.cornell.edu/brc/genomic-diversity

2 https://sites.google.com/site/africropproject/data

3 http://www.R-project.org

## Supplementary Material

FIGURE S1 **Distribution of minor alleles frequencies (MAF) estimated by within the TM pipeline for shared SNPs**.

TABLE S1 **List of SNPs shared between both datasets**.
