# Supplementary figures and images for: Genotyping-by-Sequencing SNP Identification for Crops without a Reference Genome: Using Transcriptome Based Mapping as an Alternative Strategy
Source: Front Plant Sci. 2016 Jun 15;7:777. doi: 10.3389/fpls.2016.00777 (PMC4908121; doi:10.3389/fpls.2016.00777)

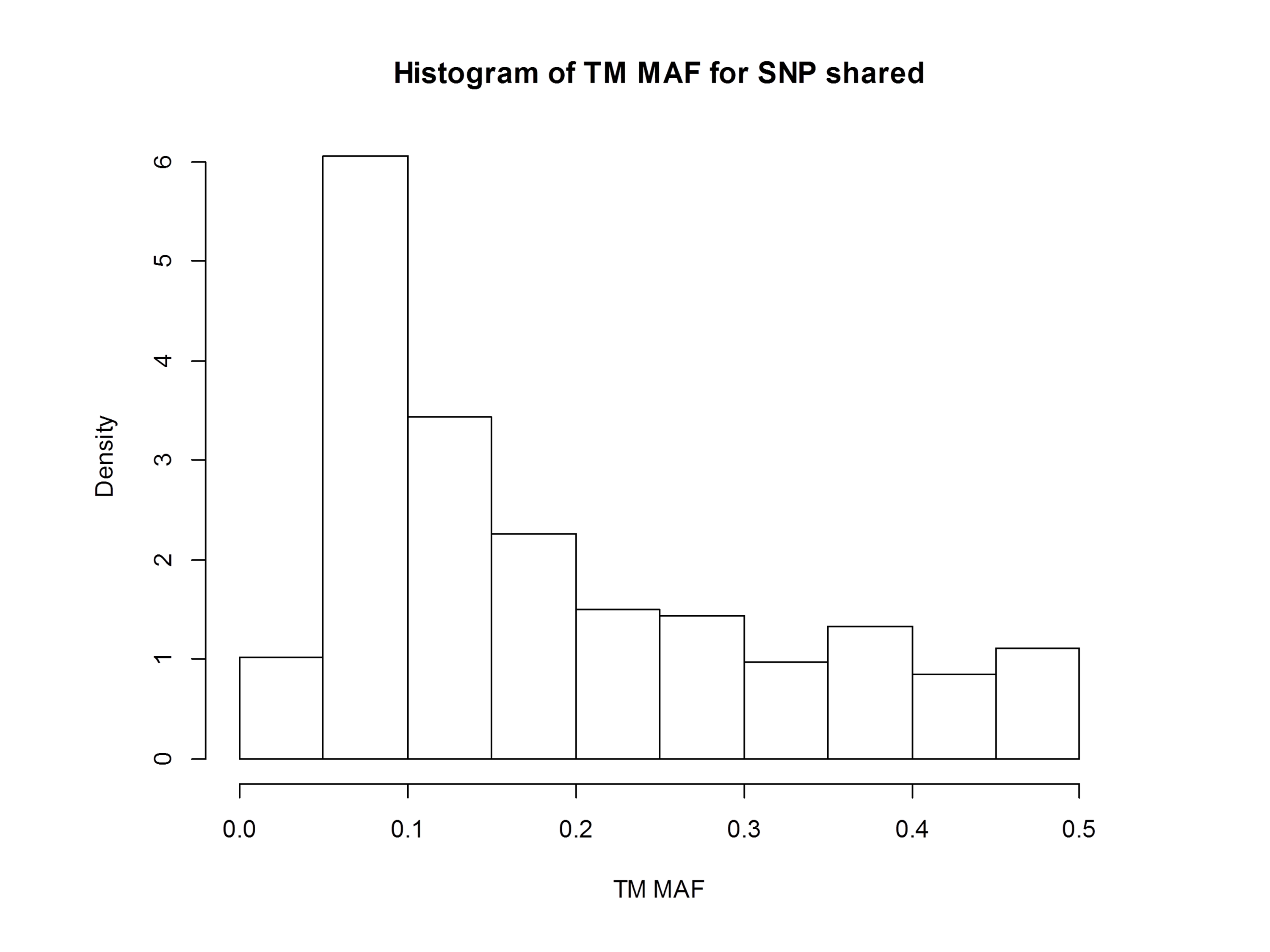

Supplement: FIGURE S1 — Distribution of minor alleles frequencies (MAF) estimated by within the TM pipeline for shared SNPs. [file Image_1.TIF]
