# Supplementary material for: Genotyping-by-Sequencing SNP Identification for Crops without a Reference Genome: Using Transcriptome Based Mapping as an Alternative Strategy
Source: Front Plant Sci. 2016 Jun 15;7:777. doi: 10.3389/fpls.2016.00777 (PMC4908121; doi:10.3389/fpls.2016.00777)
Supplement: Supplementary file 3 [file Presentation_1.PDF]

```
###################
## File preparation ##
###################

# Unzipping FastQC files
for i in *.gz; do  gunzip -d *.gz; done

# Quality control with FastQC
for i in *.fastq; do fastqc $i; done

# Demultiplex R1 files with demultadapt.py
for i in *.fastq; do python2.6 demultadapt.py -f $i -p GBS FILE_ADAPT; done  #see at the end of the file for FILE-ADAPT

# Remove adpters with Cutadapt
for i in *fastq; do cutadapt -q 20 -m 35 -o Cutadapt-$i $i; done 

# Quality filter with Filter.pl
for i in Cutadapt*.fastq; do perl Filter_Fastq_On_Mean_Quality.pl -f $i  -o Filter-$i; done




############
## Mapping ##
############

# Mapping with BWA
bwa index Transcriptome.fasta
for i in Filter-*.fastq; do pattern=${i%%.fastq}; bwa aln Transcriptome.fasta $i > ${pattern}.sai; done 
for i in *.sai; do  pattern=${i%%.sai}; bwa samse -n 3 transcriptome.fasta ${pattern}.sai ${pattern}.fastq > ${pattern}.sam; done

# Remove unmapped reads with Samtools view
for i in *.sam ; do pattern=${i%%.sam} ; samtools view -S -b -F 0x0004 $i -o ${pattern}.bam; done

# Sort and indexing reads with Samtools sort
for i in *.bam; do samtools sort $i Sort-$i; done
for i in Sort-*.bam; do samtools index $i ; done
########################
## SNP calling with GATK ##
########################

# Add a reag group with Picard-tools AddOrReplaceReadGroups
for i in  Sort-*.bam; do n=`echo $i | cut -c 6-15`; pattern=${n%%.*}; AddOrReplaceReadGroups.jar INPUT= $i OUTPUT=RG-$i RGID= ${pattern} RGLB=${pattern} RGPL= ${pattern} RGSM=${pattern} RGPU=${pattern} VALIDATION_STRINGENCY=SILENT;done

for i in RG*.bam;do samtools index $i; done
# Merge .bam with Picard-tools MergeSamFiles
MergeSamFiles.jar OUTPUT=Merge.bam $(printf "INPUT=%s " *.bam)

# Index .bam and Reference.fasta file with Samtools index et faidx
samtools index Merge.bam
samtools faidx Transcriptome.fasta
```

```

# Creation of Reference dictionary with Picard-tools CreateSequenceDictionary
-jar CreateSequenceDictionary.jar REFERENCE=Transcriptome.fasta OUTPUT= Transcriptome.dict

# Indels treatment with GATK TargetCreator
-jar GenomeAnalysisTK.jar -T RealignerTargetCreator -R Transcriptome.fasta -I Merge.bam -o
INDEL.intervals

# Realignment with GATK IndelRealigner
-jar GenomeAnalysisTK.jar -T IndelRealigner --targetIntervals INDEL.intervals -o Realign.bam
-I Merge.bam -R Transcriptome.fasta 

# SNP calling with GATK UnifiedGenotyper
-jar GenomeAnalysisTK.jar -T UnifiedGenotyper -I Realign.bam -R Transcriptome.fasta  -o
Calling.vcf -glm BOTH -stand_emit_conf 10 

# SNP filterting

-jar GenomeAnalysisTK.jar -T SelectVariants --variant Calling.vcf -R Transcriptome.fasta  -o
Biallelic.vcf -restrictAllelesTo BIALLELIC

vcftools --vcf  Biallelic.vcf --remove-indels --out TMP.vcf –recode

#filter for no more than 3 mismatches per 10 bp window
-jar GenomeAnalysisTK.jar -T VariantFiltration --variant TMP.vcf -R  Transcriptome.fasta -o
TMP.vcf --clusterSize 3 --clusterWindowSize 10 

#filter for mapping quality
-jar GenomeAnalysisTK.jar -T VariantFiltration --variant TMP.vcf -R  Transcriptome.fasta -o
TMP.vcf --filterExpression "MQ0 >= 4 && ((MQ0 / (1.0 * DP)) > 0.1)" --filterName
"HARD_TO_VALIDATE" 

#filter on SNP quality
-jar GenomeAnalysisTK.jar -T VariantFiltration --variant TMP.vcf -R  Transcriptome.fasta -o
TMP.vcf --filterExpression "QD<=6.87" --filterName "LowQD" --filterExpression "QUAL<=60.0" --
filterName "LowQUAL" 

vcftools --vcf  TMP.vcf --remove-filtered-all --out TMP-2.vcf --recode

###filtering on remaining SNPs for depth (median value) and missing data (10 %)

-jar GenomeAnalysisTK.jar -T VariantFiltration --variant TMP-2.vcf -R  Transcriptome.fasta -o
 TMP-3.vcf --filterExpression "DP<=90.0" --filterName "LowDP" 
vcftools --vcf  TMP-2.vcf --remove-filtered-all --out TMP-3.vcf --recode
vcftools --vcf  TMP-3.vcf --geno 0.9 --out TPM-final.vcf --recode


##################
## File FILE_ADAPT ##
##################

CTCC	838B1
TTCTC	838C1
GCTTA	1426B1
AACGCCT	1440A1
AGGC	1458B2
TCGTT	1458E1
```

```
TGGCTA	1511B1
TGCTGGA	1511D1
TGCA	1514B1
AGCCC	blank
CTTCCA	2747D2
AATATGC	2747E1
GATC	5710A1
ACCTAA	5710C1
ACGTGTT	5720B2
AACCGAGA	5720E1
ACTA	5722C1
GTATT	5722D2
GAGATA	5724A2
ACGACTAC	5724D1
TCAC	5726B1
ATATGT	5726E2
ATTAATT	5727B1
ACAGGGAA	5727E1
CAGA	8038B2
CTGTA	8038C2
ATGCCT	8084B2
GGTGT	8084C1
AGGAT	8094A1
ATCGTA	8094E2
ATTGGAT	8105D2
ACGTGGTA	8105E2
AACT	8106A1
ACCGT	8106E1
TATTTTT	8121A2
TAGCATGC	8121D1
ATTGA	8126A2
CATCGT	8126D1
CATAAGT	8127A1
CCATGGGT	8127E2
GCGT	8136A1
GTAA	8136B1
CTTGCTT	8146B1
AGTGGA	8146D1
CATCT	8155A1
CGCGGT	8155C1
CGCTGAT	8159B2
CGCGGAGA	8159D2
TGCGA	8165A2
GGTTGT	8165B1
ATGAAAC	8171A1
TAGGCCAT	8171C1
CCTAC	8176B1
CTATTA	8176B2
CGGTAGA	8185B1
CGTGTGGT	8185C1
CGAT	8470D1
CCAGCT	8470E1
AAAAGTT	8473A2
TGCAAGGA	8473C1
GAGGA	8482A1
GCCAGT	8482C2
CTACGGA	8487C2
```

```
GCTGTGGA	8487E1
CGCTT	8492B2
TTCAGA	8492E2
GAATTCA	8498B2
TGGTACGT	8498D1
GGAAC	8503A1
GGAAGA	8503D2
GCGGAAT	8721B2
GGATTGGT	8721D2
TCACC	8725B2
TAGGAA	8725C1
GAACTTC	8726B2
TCTCAGTC	8726C1
GTCAA	8727C2
GTACTT	8727D1
TAGCGGA	8730A1
GTGAGGGT	8730E1
CTAGC	8731A1
GCTCTA	8731C1
GGACCTA	8732B2
CCGGATAT	8732D1
TAATA	8734A2
GTTGAA	8734B2
TCGAAGA	8736A1
TATCGGGA	8736C2
ACAAA	8739D2
CCACAA	8739E1
GTCGATT	8743A1
CGCCTTAT	8743B2
TACAT	8744A1
TAACGA	8744B1
TCTGTGA	11356C1
TTCCTGGA	11356B2
* noindex


#####################
## R script for performing PCA##
#####################

library(SNPRelate)
TM.fn <- "TM.vcf"
snpgdsVCF2GDS(TM.fn,"TM.gds",method="biallelic.only")
snpgdsSummary("TM.gds")
genofile_TM<-openfn.gds("TM.gds")

uneak.fn <- "UNEAK.vcf"
snpgdsVCF2GDS(uneak.fn,"uneak.gds",method="biallelic.only")
snpgdsSummary("uneak.gds")
genofile_U<-openfn.gds("uneak.gds")
TM.id <- read.gdsn(index.gdsn(genofile, "sample.id"))
UN.id <- read.gdsn(index.gdsn(genofile_U, "sample.id"))


# run PCA transricptome
pop.trans<-c(rep("838",2),rep("11356",2),rep("1458",2),rep("1511",2),rep("1514",1),rep("2747"
,2),rep("5710",2),rep("5720",2),rep("5722",2),rep("5724",2),rep("5726",2),rep("5727",2),rep(
```

```
"8038",2),rep("8084",2),rep("8094",2),rep("8105",2),rep("8106",2),rep("8121",2),rep("8126",2
),rep("8127",2),rep("8136",2),rep("8146",2),rep("8155",2),rep("8159",2),rep("8165",2),rep(
"8171",2),rep("8176",2),rep("8185",2),rep("8470",2),rep("8473",1),rep("8482",2),rep("8487",2
),rep("8492",2),rep("8498",1),rep("8503",2),rep("8721",2),rep("8725",1),rep("8726",2),rep(
"8727",2),rep("8730",2),rep("8731",2),rep("8732",2),rep("8734",2),rep("8736",1),rep("8739",2
),rep("8744",2))

pca.trans <- snpgdsPCA(genofile_TM, autosome.only=FALSE) 
tab.trans<- data.frame(sample.id = pca.trans$sample.id,
pop = factor(pop.trans)[match(pca.trans$sample.id, TM.id)],
EV1 = pca.trans$eigenvect[,1], 
EV2 = pca.trans$eigenvect[,2],
stringsASFactors=FALSE)
country<-c("Mali","Mauritania","Niger","Senegal","Sudan","Tchad")
plot(tab.trans$EV1, tab.trans$EV2, xlab="PC2-6.5%", ylab="PC1-8% ",pch=18)
legend("bottomright", legend=country, pch=18)
pc.trans.percent <- 100 * pca.trans$eigenval[1:16]/sum(pca.trans$eigenval)
pc.trans.percent
lbls <- paste("PC", 1:4, "\n", format(pc.trans.percent[1:4], digits=2), "%", sep="")
pairs(pca.trans$eigenvect[,1:4], col=col1, labels=lbls)

# run PCA uneak
pop.uneak<-c(rep("11356",2),rep("1458",2),rep("1511",2),rep("1514",1),rep("2747",2),rep(
"5710",2),rep("5720",2),rep("5722",2),rep("5724",2),rep("5726",2),rep("5727",2),rep("8038",2
),rep("8084",2),rep("8094",2),rep("8105",2),rep("8106",2),rep("8121",2),rep("8126",2),rep(
"8127",2),rep("8136",2),rep("8146",2),rep("8155",2),rep("8159",2),rep("8165",2),rep("8171",2
),rep("8176",2),rep("8185",2),rep("838",2),rep("8470",2),rep("8473",1),rep("8482",2),rep(
"8487",2),rep("8492",2),rep("8498",1),rep("8503",2),rep("8721",2),rep("8725",1),rep("8726",2
),rep("8727",2),rep("8730",2),rep("8731",2),rep("8732",2),rep("8734",2),rep("8736",1),rep(
"8739",2),rep("8744",2))
pca.uneak <- snpgdsPCA(genofile_U,sample.id =u.id, autosome.only=FALSE) 
tab.uneak<- data.frame(sample.id = pca.uneak$sample.id,
pop = factor(pop.uneak)[match(pca.uneak$sample.id, UN.id)],
EV1 = pca.uneak$eigenvect[,1], # the first eigenvector
EV2 = pca.uneak$eigenvect[,2],
stringsASFactors=FALSE)
plot(tab.uneak$EV1, tab.uneak$EV2, col=col1u,xlab="PC2-6.5%", ylab="PC1-8% ",pch=18)
legend("topright", legend=pays, pch=18)
pc.uneak.percent <- 100 * pca.uneak$eigenval[1:16]/sum(pca.uneak$eigenval)
pc.uneak.percent
lbls <- paste("PC", 1:4, "\n", format(pc.uneak.percent[1:4], digits=2), "%", sep="")
pairs(pca.uneak$eigenvect[,1:4], col=col1u, labels=lbls)
```
